# Longitudinal Associations Between Fruit and Vegetable Prescription Program Participation and Pediatric Blood Pressure and Weight Status

**DOI:** 10.3390/nu18213476

**Published:** 2026-10-23

**Authors:** Amy Saxe-Custack, Diana Haggerty, Jenny LaChance, Sarah Egan, David Todem

**Affiliations:** 1 Charles Stewart Mott Department of Public Health, Michigan State University–Hurley Children’s Hospital Pediatric Public Health Initiative, Room 209, 200 E 1st Street, Flint, MI 48502, USA; hagger39@msu.edu (D.H.); lachan14@msu.edu (J.L.); egansar2@msu.edu (S.E.); 2 Department of Epidemiology and Biostatistics, College of Human Medicine, Michigan State University, East Lansing, MI 48824, USA; todem@msu.edu

**Keywords:** pediatrics, blood pressure, produce prescriptions, primary health care, body mass index

## Abstract

**Background/Objectives**: This study examined whether engagement in a pediatric fruit and vegetable prescription program (FVPP) offering one $15 prescription during office visits influenced health, hypothesizing that youth improve blood pressure (BP) and weight status after one year. **Methods**: This non-controlled longitudinal intervention trial included data from caregiver–child dyads at one pediatric clinic (child age 8–16 years). Correlated data models evaluated average longitudinal trends of study endpoints. Causal inference analyses for longitudinal data estimated time-varying effects of the FVPP on outcomes. **Results**: In total, 304 dyads enrolled. Youth reduced mean body mass index (BMI) percentile (*p* = 0.02), systolic BP (*p* < 0.001), and diastolic BP (*p* = 0.01). Youth also reduced the odds of elevated systolic BP (*p* < 0.001), diastolic BP (*p* = 0.004), and overall BP (*p* = 0.003). Children who redeemed ≥1 prescription reduced BMI percentile compared to counterparts who did not redeem (*p* = 0.02). Children who redeemed ≥1 prescription between baseline and 6 months reduced odds of obesity or elevated systolic BP at 12 months, with a relative odds ratio of 0.47 (*p* = 0.01) or 0.51 (*p* = 0.05), compared to peers who did not redeem. **Conclusions**: The results suggest that pediatric FVPP engagement may contribute to improvements in key health outcomes, underscoring the need for further investigation.

## 1. Introduction

Fruit and vegetable prescription programs, which typically offer prescriptions distributed by healthcare providers that patients can exchange for fruits and vegetables at local food retailers, are increasingly available across the United States. When given to adults, studies have demonstrated associations between participation in fruit and vegetable prescription programs and improvements in dietary patterns, food security, and clinical outcomes [1,2,3]. However, the effectiveness of pediatric fruit and vegetable prescription programs in improving health metrics among youth is unclear [4,5].

Pediatric clinics throughout Flint, Michigan, a low-income urban community, are currently offering $15 fresh produce prescriptions to pediatric patients. These prescriptions are provided to all pediatric patients during regular office visits to support the purchase and consumption of fruits and vegetables among youth. Patients can redeem prescriptions at a downtown farmers’ market or local mobile market with home delivery options (flintfresh.org). Previous research has suggested that access to this fruit and vegetable prescription program (FVPP) is associated with improvements in food security and child dietary behaviors [6,7]. Moreover, a recent cross-sectional study demonstrated that youth with access to this FVPP had healthier blood pressure and physical activity levels compared to youth who did not have access [8]. This study examined the impact of the pediatric FVPP on youth blood pressure and weight status after one year of program exposure.

## 2. Materials and Methods

### 2.1. Intervention

Michigan’s first FVPP for children was introduced in February 2016 at Hurley Children’s Center, a residency-training pediatric clinic that shares a building with a year-round farmers’ market. When designing the program, researchers, pediatricians, and clinic staff collaborated to develop a program that was easy to administer within a busy pediatric office as well as universal in its messaging about the importance of regular consumption of fruits and vegetables. Prescriptions were added to the electronic medical record (EMR) system to simplify distribution and enable tracking of prescriptions within the EMR. Pediatricians ordered fruit and vegetable prescriptions through the EMR system and distributed prescriptions (printed on prescription paper) at the end of all office visits. Patients had up to 90 days to redeem prescriptions for $15 of fresh fruits and vegetables either at a local farmers’ market or a mobile market that offered free home delivery. Once prescriptions were redeemed, produce vendors returned prescriptions to the research team, allowing researchers to reimburse the vendors and track prescription usage.

In response to the FVPP’s success at Hurley Children’s Center (Clinic 1), the identical program was expanded to Akpinar Children’s Clinic (Clinic 2) in 2018 and to Mott Children’s Health Center (Clinic 3) in 2021. A related cross-sectional study that compared caregiver–child dyads with high exposure to the FVPP at Hurley Children’s Center (Clinic 1) and Akpinar Children’s Clinic (Clinic 2) to dyads with no/low exposure at Mott Children’s Health Center (Clinic 3) demonstrated that longer exposure to the FVPP was associated with healthier blood pressure among youth [8].

### 2.2. Design and Sample

The sample for this non-controlled longitudinal intervention trial consisted of caregiver–child dyads at Mott Children’s Health Center (Clinic 3) as the FVPP was introduced there. A consecutive sample of caregivers with children aged 8–16 years was eligible to enroll in the current study. Caregiver–child dyads were excluded if they did not speak English; the caregiver was not the child’s legal guardian; the child did not provide assent; the child was a previous patient at Clinic 1 or 2; or a sibling was enrolled in the study (one dyad eligible per household). Children who had pre-existing metabolic conditions, were overweight or obese, or experienced hypertension or other cardiovascular conditions were eligible to participate in the current study.

After providing consent and assent, trained research assistants explained to caregivers and children how to complete the baseline surveys. Dyads used iPads in the clinic to individually answer questions in the baseline survey related to demographics, diet, food security, and physical activity using a secure online platform (Research Electronic Data Capture or REDCap). During the baseline appointment, research assistants also followed standardized procedures to measure child weight and height and obtain systolic blood pressure (SBP) and diastolic blood pressure (DBP) measures and measurement dates from patient medical charts. Approximately 6 and 12 months after baseline data collection, caregivers and children completed follow-up surveys either at the pediatric clinic or virtually. Survey data completion, weight and height assessments, and blood pressure measurements extracted from medical charts occurred at baseline/enrollment, 6-month follow-up, and 12-month follow-up.

To improve the precision of the trial estimates, data from Clinic 3 were supplemented with data collected cross-sectionally at Clinics 1 and 2 to estimate variance in models as described in the section on statistical analysis. Data collection methods and instruments were identical across each clinic but were collected only at baseline at Clinics 1 and 2. Because children at Clinics 1 and 2 did not contribute longitudinal data, the reported results only include outcomes for children from Clinic 3. All data were collected from March 2021 through May 2023. The Michigan State University Institutional Review Board approved this study, and all participants provided informed written consent. The study was registered through clinicaltrials.gov [ID: NCT04767282] on 23 February 2021.

Based on previous findings [9], the sample of 304 caregiver–child dyads from Clinic 3 gives more than 90% power to detect an improvement in the mean intake of vegetables of 0.18 cups over 12 months, assuming a temporal correlation of 0.2 and a two-sided 5% significance level using an underdispersed, correlated Poisson model with a timepoint dispersion parameter of 0.82.

### 2.3. Measures

#### 2.3.1. Child Weight Status

Research assistants collected children’s weight and height without shoes or heavy outer clothing. On each occasion (baseline, 6, and 12 months), they obtained and recorded two measures, and the average of the two measures was used in the analyses. Weight was collected on a digital platform scale accurate to 200 kg, and measures were rounded to the nearest 0.2 kg. Height was collected using a portable stadiometer, and measures were rounded to the nearest 0.1 cm. When research assistants were unable to measure youth weight and height, data were extracted from medical charts. Child body mass index (BMI) was computed using child weight and height measures (weight (kg)/[height (m)]^2^). Then, researchers categorized child BMI into percentiles by sex and age to be used as a marker of overweight and obesity [10]. Caregiver BMI was determined using self-reported weight and height and used as a moderating variable for child BMI percentile.

#### 2.3.2. Child Blood Pressure

Trained research assistants extracted systolic and diastolic blood pressure values from medical charts. Approximately 92% of blood pressure measurements were taken by trained clinic staff who followed standardized procedures for pediatric settings. For each measurement, the child’s date of birth, measurement date, biological sex, height (cm), and systolic and diastolic blood pressure values (mmHg) were entered into the Baylor College of Medicine Age-Based Pediatric Blood Pressure Calculator. The tool calculated systolic and diastolic blood pressure percentiles, Z-scores, and height percentiles based on normative reference data from the 2017 American Academy of Pediatrics (AAP) Clinical Practice Guidelines [11]. Blood pressure values were then categorized during data analysis using criteria from the 2017 AAP pediatric hypertension guidelines [12].

#### 2.3.3. Child Dietary Patterns

Dietary patterns were measured using the 41-item Block Kids Food Screener (BKFS) [13]. Dietary analysis, using the Block Online Analysis System, provided nutrient estimates and the number of servings by food groups. These data were used to determine mean daily intake of whole fruits, total fruits, and total vegetables.

#### 2.3.4. Child-Reported Food Security

Child food security status was measured in children aged 12 years and older using the Self-Administered Food Security Survey Module for Youth [14]. Food security status was assigned a score based on the number of positive responses to nine questions. Scores ranged from 0 to 9 (0–1 = high/marginal food security; 2–5 = low food security; 6–9 = very low food security).

#### 2.3.5. Household Food Security

Caregivers reported household food security using the US Household Food Security Module: Six Item Short Form (National Center for Health Statistics) [15]. The sum of affirmative responses to six questions served as the household’s raw score. Food security status was assigned based on a calculated raw score (0–1 = high/marginal food security; 2–4 = low food security; 5–6 = very low food security).

#### 2.3.6. Child Physical Activity

Physical activity was measured with the 8-question Patient-Reported Outcomes Measurement Information System (PROMIS) Physical Activity questionnaire for children and adolescents. Questions asked how many days per week children engaged in various types of physical activity (1 = no days; 2 = 1 day; 3 = 2–3 days; 4 = 4–5 days; 5 = 6–7 days). Scores ranged from 8 to 40.

#### 2.3.7. Demographics

At baseline, caregivers self-reported age, education, race, weight and height, city of residence, household income, and household participation in food assistance programs. With help from caregivers and research assistants, youth self-reported age, sex, race, and city of residence.

#### 2.3.8. Prescription Redemption

Fruit and vegetable prescription redemption was a time-dependent variable that counted the number of redeemed prescriptions between baseline and 6 months, and between 6 months and 12 months. This variable was dichotomized to binary, taking value 1 if the participant redeemed at least 1 fruit and vegetable prescription in the 6-month timeframe, and 0 otherwise. Supplemental Figure S1 demonstrates the hypothetical relationships of variables over time.

### 2.4. Analysis

Two longitudinal analyses of data collected at baseline, 6 and 12 months were conducted. One analysis describes the average longitudinal trends of primary and secondary study endpoints, and the other evaluates the effects of prior fruit and vegetable prescription redemptions on these endpoints. Estimates and contrasts representing these trends and effects were generated for study participants in Clinic 3. To improve efficiency in detecting differences from baseline, data from Clinics 1 and 2 were leveraged to estimate baseline error variances. Missing data were handled based on available case analysis, consistent with a less stringent missing at random mechanism [16]. Specifically, subjects with incomplete data profiles were included in the analysis. Patterns of missing data are available in Supplemental Table S1. This approach is consistent with our inclusion of subjects in Clinics 1 and 2 in the analysis, even though attrition at these clinics was by design. When missing data resulted from unavailable research assistant measurements, the clinic measurements recorded in medical records were imputed to the unobserved values. The statistical model was then extended to account for differences in measurement methods by allowing the mean and covariance terms to depend on the nature of the measurement.

Correlated data models were conducted to evaluate the average longitudinal trend of primary and secondary endpoints. Specifically, a linear mixed model (continuous endpoints, e.g., total fruit consumption) [17] or a generalized linear mixed model (binary endpoints, e.g., obesity) [17] with a subject random effect was used to describe the average profiles of study endpoints across time. To protect against any misspecification of the underlying correlation model, sandwich-based covariance estimators were considered to generate consistent standard errors for the fixed effects parameter estimates. The basic mean model did not assume that changes over time followed a straight-line (linear) pattern, allowing for more complex or non-linear trends. Longitudinal trends were then evaluated by contrasting 6 months against baseline (initial pattern) and 12 months against 6 months (follow-up pattern), using differences for continuous endpoints (e.g., physical activity) and odds ratios for binary endpoints (e.g., having elevated blood pressure).

Longitudinal analyses were further conducted to estimate the total effects of the FVPP on study outcomes, focusing on prescription redemption. By total effects, we refer to both the indirect effect of the FVPP exposure at a given period on a future outcome through future intermediate FVPP exposures and its direct effect not mediating through these future exposures. When there are no intermediate exposures, the total effect reduces to the direct effect. Because our analytic sample involves a baseline and two follow-up visits (6 and 12 months), two total effects (lag 1 and lag 2) were considered. Lag 1, the short-term or immediate effect, corresponds to the effects of the FVPP exposure during the last 6 months on the endpoints (that is, exposure between baseline and 6 months on the 6-month response, or the exposure between 6 months and 12 months on the 12-month response). Lag 2, the long-term or delayed effect, corresponds to the effects of the FVPP exposure between baseline and 6 months on the response at 12 months. To control for measured confounders at any timepoint, the basic mean model included prior values of the exposure and outcome, in addition to time-varying and time-invariant baseline covariates. The lag effects, representing associations from the classical conditional model, were then estimated using generalized estimating equations (GEEs) [18,19] with sequential conditional mean models (SCMMs) [20] for the repeated outcomes and independence working correlations.

All analyses were performed with the use of SAS software, version 9.4 (SAS Institute) [21]. Further details of the statistical analysis, including specification of the models, are provided in the Supplementary Materials.

#### Sensitivity Analysis

We conducted a sensitivity analysis to understand the impact of replacing research assistant measured height and weight with clinic data. Specifically, we evaluated the child BMI outcomes without replacing missing research assistant measured height and weight with clinic-recorded data for the longitudinal trends and effects models of prescription redemption. Results of this analysis are provided in the Supplementary Materials.

## 3. Results

A total of 304 caregiver–child dyads (608 participants) enrolled in the study at Mott Children’s Health Center (Clinic 3). Mean age of youth was 11.98 years, and most reported race as African American (68%). The majority of households resided in Flint (73.58%) and participated in the Supplemental Nutrition Assistance Program/SNAP (63.67%). Participant characteristics and comparisons to dyads at Clinics 1 and 2 are provided in Supplemental Tables S2–S4.

As shown in Table 1, there was a significant reduction in mean BMI percentile among youth participants at Clinic 3 during the study period (*p* = 0.02), controlling for baseline confounders and baseline subject random effects. The 2.28% reduction in BMI percentile occurred between baseline and 12 months (*p* = 0.004). No significant change was observed between baseline and 6 months (*p* = 0.07) or between 6 and 12 months (*p* = 0.87).

Youth participants experienced a reduction in SBP (*p* < 0.001) and DBP (*p* = 0.01) throughout the study period, controlling for baseline confounders and subject random effects. Compared to baseline, participants reduced SBP percentiles at 6 months by 3.99% (*p* = 0.04) and at 12 months by 7.5% (*p* < 0.001). The reduction in DBP percentiles occurred only at 12 months with a 5.12% reduction compared to baseline (*p* = 0.003).

Youth at Clinic 3 reduced their odds of elevated SBP (*p* < 0.001), DBP (*p* = 0.004) and overall blood pressure (BP) (*p* = 0.003) throughout the study period, controlling for baseline confounders and baseline subject random effects. Compared to baseline, youth odds of elevated SBP at 6 months are multiplied by 0.44 (*p* = 0.001) and at 12 months by 0.48 (*p* = 0.001). The reduction in odds of elevated DBP and overall BP were observed only at 12 months, with a relative odds ratio of 0.46 (*p* < 0.001) and 0.52 (*p* < 0.001) compared to baseline, respectively.

As shown in Table 2, long-term effects of redemption were observed for both endpoints representing child weight status (BMI percentile and obesity) and elevated SBP. Specifically, children who redeemed at least one prescription between baseline and 6 months reduced their BMI percentile by 7.48 (*p* = 0.02) at 12 months compared to their counterparts who did not redeem a prescription during the same timeframe. Likewise, children who redeemed at least one prescription between baseline and 6 months reduced their odds of obesity or elevated SBP at 12 months, with a relative odds ratio of 0.47 (*p* = 0.01) or 0.51 (*p*-value = 0.05) compared to their peers who did not redeem a prescription during the same timeframe.

Among youth participants at Clinic 3, physical activity improved at 6 months and 12 months compared to baseline (*p* = 0.002), controlling for baseline confounders and individual propensity (random subject effects) for engaging in physical activity (Table 3). Compared to baseline, youth improved physical activity at 6 months by 2.46 points (*p* = 0.001) and at 12 months by 1.89 points (*p* = 0.007). There was no improvement between 6 and 12 months *(p* = 0.44). Mean daily intake of total fruits also increased during the study period (*p* = 0.04). Youth increased mean daily intake of total fruits at 6 months by 0.22 cups compared to baseline (*p* = 0.04). There was no improvement in mean daily intake of fruits between 6 and 12 months (*p* = 0.68). Changes related to other dietary factors and food security were not significant (Supplemental Table S5).

### Sensitivity Analyses

Results for the sensitivity analysis did not differ qualitatively from those of the analysis that included clinic-replaced data for the longitudinal trend in BMI percentile and obesity and the lag effects of prescription redemptions on BMI percentile and obesity (Supplemental Tables S6 and S7). Specifically, a similar pattern of change in BMI percentile for the entire study period was observed, with a significant contrast between 12 months and baseline. A significant longitudinal change in obesity was not found in either analysis. The lag 2 effect of prescription redemption remained significant. The diagram highlighting the lag 1 and 2 causal effects are depicted in Figure S2.

## 4. Discussion

This is the first longitudinal study in which youth engagement in a pediatric FVPP, available to all patients, was associated with improvements in clinical markers of health among children. Related cross-sectional research previously suggested that patients who were exposed to this pediatric FVPP recorded healthier blood pressure and more desirable physical activity patterns than patients who were not exposed [8]. The current study confirms these findings and further suggests that program engagement may be associated with improvements in BMI percentile among pediatric patients.

Significant improvements in blood pressure were found among young participants in this study. Hypertension in pediatric patients is not only related to subclinical atherosclerosis and target organ damage in youth but can also elevate cardiovascular disease risk later in life [22]. Unmanaged hypertension is pervasive and devastating, particularly among African Americans who, compared to individuals in other racial/ethnic groups, experience earlier onset of high blood pressure [23] and exhibit lower compliance with antihypertensive medication therapy [24]. The current study showed improvements in systolic and diastolic blood pressure over 12 months of exposure, suggesting that this FVPP may have important clinical impacts within the first year of program implementation. Moreover, the observed improvements in blood pressure and weight status at 12 months were associated with redemption of produce prescriptions during the first 6 months of study.

Hypertension, while formerly considered an adult diagnosis, is increasingly common among pediatric patients [25]. This trend is associated with various factors, including poor diets in childhood and reduced physical activity, which increase childhood obesity risk [26]. Early onset of hypertension in pediatric patients can lead to negative health outcomes that persist into adulthood [22,27]. The rise in pediatric hypertension represents an important target for primary care preventative medicine to reduce morbidity and mortality associated with the long-term effects of hypertension. The current model of FVPP was designed with pediatricians to facilitate easy implementation within busy clinics while providing a valuable resource to support healthy lifestyles. The current study provides evidence to suggest that pediatric FVPPs are an important resource to support primary care providers in their efforts to actively prevent and manage pediatric hypertension.

Although dietary patterns initially improved among youth exposed to the FVPP, only increases in mean daily intake of fruits were significant. This finding supports previous research that suggests youth who are newly introduced to this FVPP tend to purchase primarily fruits during the initial months of the program, with changes in vegetable intake requiring longer exposure [6,28]. Results of the current study did not demonstrate an association between prescription exposure and changes in dietary patterns or food security, contradicting previous research [6,7,29,30]. It is important to note that baseline data collection occurred while the Child Tax Credit (CTC) was substantially increased (July 2021 through December 2021). Research examining the impacts of the expanded CTC has shown that the initiative reduced food insufficiency, caused families to have more funds to purchase food, improved healthy eating, and offset the effects of inflation on family budgets [31,32,33,34]. Therefore, the expanded CTC, available only during baseline data collection, likely inflated baseline food security and dietary measures, limiting the calculated impact on diet.

In addition to the CTC, COVID-related changes in the broader socioeconomic context over the study period may have influenced fruit and vegetable prescription program engagement due to declines in healthcare utilization. Research has shown sharp reductions in pediatric healthcare utilization during the pandemic, which undoubtedly resulted in fewer prescriptions distributed [35]. In recent years, as pediatric patients return to in-person office visits, both prescription distribution and redemption rates have markedly increased.

Although associations between exposure to the FVPP and changes in diet and food security were not significant, the pediatric FVPP may have influenced health through various mechanisms that were not assessed in the current study. For example, related research has shown that caregiver perceptions of pediatric healthcare were improved among parents who engaged in the current pediatric FVPP with their children [36]. These findings underscore the potential of produce prescription programs designed for youth to improve or strengthen caregiver–provider relationships. Similarly, pediatricians and clinic staff have suggested that the FVPP serves as an incentive for families to regularly visit their primary care provider, rather than urgent care or emergency rooms [37]. These observed patterns indicate that integrating resource-based interventions into pediatric primary care may strengthen both family engagement and perceived quality of healthcare, which are critical in improving health equity and partnerships between providers and families [38]. In addition to improving direct access to fresh foods, pediatric FVPPs could function as broad behavioral and social interventions that positively influence health through multiple pathways, including greater engagement with healthcare services, increased health awareness, family-level behavioral changes, or improved interactions with other supportive programs.

The current study has limitations. First, the primary outcome of the original study was fruit and vegetable intake and may be underpowered for the secondary outcomes presented here. Additionally, the sample was specific to one geographic location, limiting generalizability. Engagement in COVID-related relief programs was not measured, and this may have influenced baseline dietary patterns and food security. Next, the authors cannot rule out residual and unmeasured confounding that may have independently influenced blood pressure and body weight, particularly that participants who actively engaged with the FVPP may have differed systematically from those who did not in terms of motivation to improve health, family support, or health literacy. Finally, the findings of our analysis may be biased due to nonresponse selection in the direction of informative missingness.

## 5. Conclusions

This is the first longitudinal study to suggest that pediatric FVPP engagement may be associated with improvements in key health outcomes. Findings fill important gaps in evidence regarding clinical effects of pediatric FVPPs.

## Figures and Tables

**Table 1:** Adjusted estimates and confidence intervals for contrasts evaluating the longitudinal trend for clinical outcomes in Mott Children’s Health Center patients in Flint, Michigan, 2021–2023.

Clinical Outcomes	Longitudinal Comparisons ^a^			
	6 Months Versus Baseline Estimate (CI)	12 Months Versus Baseline Estimate (CI)	12 Months Versus 6 Months Estimate (CI)	Overall Trend *p*-Value ^b^
**Child weight status**				
BMI percentiles (%)	−2.13 (−4.39, 0.14)	−2.28 (−3.85, −0.72)	−0.15 (−2.01, 1.70)	0.02
Obesity (BMI% > 95%) ^c^	0.70 (0.46, 1.08)	0.83 (0.65,1.05)	1.17 (0.82, 1.69)	0.19
**Child SBP status**				
SBP percentile (%)	−3.99 (−7.85, −0.13)	−7.50 (−11.04, −3.96)	−3.51 (−8.00, 0.98)	<0.001
Elevated SBP (SBP% > 90%) ^c^	0.44 (0.27, 0.73)	0.48 (0.31, 0.74)	1.10 (0.62, 1.94)	<0.001
**Child DBP status**				
DBP percentile (%)	−1.24 (−4.93, 2.45)	−5.12 (−8.46, −1.78)	−3.88 (−7.84, 0.09)	0.01
Elevated DBP (DBP% > 90%) ^c^	0.83 (0.54, 1.29)	0.46 (0.29, 0.72)	0.55 (0.33, 0.92)	0.004
**Elevated overall BP** ^c,d^	0.73 (0.48, 1.10)	0.52 (0.36, 0.76)	0.72 (0.46, 1.12)	0.003

BMI: body mass index, SBP: systolic blood pressure, DBP: diastolic blood pressure, BP: blood pressure. ^a^ The contrast estimates of trend are generated by controlling the following confounders: child’s age, child’s sex, child’s race (African American versus others), caregiver’s education, caregiver’s age (age < 38 years vs. age ≥ 38 years), caregiver’s BMI (BMI < 30, BMI ≥ 30, unspecified), participation of any member of the household in any supplemental nutrition assistance program or free/reduced school lunch (yes vs. no), city of residence (Flint vs. other) and caregiver’s overall perceptions of child healthcare (score < 90 vs. score ≥ 90). ^b^ *p*-values are derived from F test statistics. ^c^ Estimates on the odds ratio scale. ^d^ Normal BP: <90th percentile for children under 13 and Normal BP: <120/<80 mm Hg for children 13 and above.

**Table 2:** Effects of prescription redemption on future clinical outcomes (estimates, confidence intervals, and *p*-values) in Mott Children’s Health Center patients in Flint, Michigan, 2021–2023.

Clinical Outcomes	Adjusted Longitudinal Associations ^a^			
	Lag 1 (Short Term)		Lag 2 (Long Term)	
	Estimate (CI)	*p*-Value ^b^	Estimate (CI)	*p*-Value ^b^
**Child weight status**				
BMI percentile (%)	−1.85 (−7.55, 3.85)	0.52	−7.48 (−1.78, −1.17)	0.02
Obesity (BMI% > 95%) ^c^	0.62 (0.34, 1.14)	0.13	0.47 (0.26, 0.84)	0.01
**Child SBP status**				
SBP percentile (%)	1.96 (−2.56, 6.48)	0.39	−4.49 (−10.70, 1.73)	0.16
Elevated SBP (SBP% > 90%) ^c^	0.88 (0.50, 1.55)	0.66	0.51 (0.26, 1.00)	0.05
**Child DBP status**				
DBP percentile (%)	−0.97 (−5.39, 3.46)	0.67	−3.49 (−8.71, 1.73)	0.19
Elevated DBP (DBP% > 90%) ^c^	0.86 (0.51, 1.46)	0.57	0.77 (0.44, 1.37)	0.38
**Elevated overall BP** ^c,d^	0.85 (0.53, 1.35)	0.49	0.58 (0.32, 1.04)	0.07

BMI: body mass index, SBP: systolic blood pressure, DBP: diastolic blood pressure, BP: blood pressure. ^a^ Estimates of associations between FVPP exposure for lag 1 and 2 are computed, taking into account the presence of time-dependent confounding by controlling for prior exposures, prior outcomes, prior time-varying and non-varying (baseline) covariates. ^b^ *p*-values are generated using z tests from GEE models. ^c^ Effect estimates of associations are on the odds ratio scale. ^d^ Normal BP: <90th percentile for children under 13 and Normal BP: <120/<80 mm Hg for children 13 and above.

**Table 3:** Adjusted estimates and confidence intervals for contrasts evaluating longitudinal trends for physical activity, food security and fruit and vegetable intake in Mott Children’s Health Center patients in Flint, Michigan, 2021–2023.

Physical Activity, Food Security and Fruit and Vegetable Intake	Longitudinal Comparisons ^a^			
	6 Months Versus Baseline Estimate (CI)	12 Months Versus Baseline Estimate (CI)	12 Months Versus 6 Months Estimate (CI)	Overall Trend *p*-Value ^b^
**Physical activity**				
Raw score	2.46 (1.01, 3.91)	1.89 (0.51, 3.27)	−0.57 (−2.04, 0.89)	0.002
**Food security perception**				
Youth high food security	0.84 (0.47, 1.51)	0.69 (0.41, 1.16)	0.82 (0.44, 1.53)	0.38
Household high food security	0.86 (0.60, 1.23)	0.91 (0.62, 1.32)	1.06 (0.70, 1.59)	0.69
**Mean daily intake of fruits and vegetables (cups)**				
Total fruits	0.22 (0.01, 0.43)	0.17 (−0.01, 0.35)	−0.05 (−0.29, 0.19)	0.04
Total whole fruits	0.14 (0.02, 0.26)	0.05 (−0.06, 0.16)	−0.09 (−0.23, 0.05)	0.08
Total vegetables	0.12 (−0.05, 0.29)	−0.00 (−0.14, 0.13)	−0.12 (−0.31, 0.06)	0.34
Vegetable excl. potato/legumes	0.08 (−0.04, 0.21)	−0.02 (−0.11, 0.08)	−0.10 (−0.23, 0.03)	0.30
Total legumes	0.01 (−0.01,0.03)	−0.01 (−0.02, 0.01)	−0.02 (−0.04, 0.01)	0.29
Other fruits	0.03 (−0.01, 0.07)	0.01 (−0.02, 0.04)	−0.02 (−0.06, 0.01)	0.26
Total fruit juice	0.06 (−0.07, 0.18)	0.11 (0.001, 0.227)	0.06 (−0.08, 0.19)	0.14

^a^ Contrast estimates of trend are generated by controlling the following confounders: child’s age, child’s sex, child’s race (African American versus others), caregiver’s education, caregiver’s age (age < 38 years vs. age ≥ 38 years), caregiver’s BMI (BMI < 30, BMI ≥ 30, unspecified), participation of any member of the household in any supplemental nutrition assistance program or free/reduced school lunch (yes vs. no), city of residence (Flint vs. other) and caregiver’s overall perceptions of child healthcare (score < 90 vs. score ≥ 90). Confidence intervals are generated using sandwich-based standard errors. ^b^ *p*-values are derived from F test statistics.

## Data Availability

The data presented in this study are available on request from the corresponding author.

## Supplementary Materials

The following supporting information can be downloaded at https://www.mdpi.com/article/10.3390/nu18213476/s1. Table S1: Missing data patterns among Mott Children’s Health Center participants in Flint, Michigan, 2021–2023; Table S2: Characteristics of study participants for clinics with an established fruit and vegetable prescription program (Clinics 1 and 2) compared to the newly initiated intervention clinic (Clinic 3) in Flint, Michigan, 2021–2023; Table S3: Means and standard deviations, and analytic sample sizes for food security, physical activity, fruit and vegetable intake, and prescription exposure across time in 3 pediatric clinics in Flint, Michigan, 2021–2023; Table S4: Means and standard deviations, and analytic sample sizes for clinical outcomes among patients from 3 pediatric clinics in Flint, Michigan, 2021–2023; Table S5: Analysis evaluating the lag 1 and 2 effects of prescription redemption on physical activity, food security and fruit and vegetable intake in Mott Children’s Health Center patients in Flint, Michigan, 2021–2023; Table S6: Sensitivity analysis for contrasts evaluating the longitudinal trend for Body Mass Index (BMI) percentile (mean difference) and obesity (odds ratios); adjusted estimates and confidence intervals in Mott Children’s Health Center patients in Flint, Michigan, 2021–2023; Table S7: Sensitivity analysis on effects evaluating the lag 1 and 2 effects of prescription redemption on Body Mass Index (BMI) percentiles and obesity (estimates, confidence intervals in parenthesis and *p*-values) in Mott Children’s Health Center patients in Flint, Michigan, 2021–2023; Figure S1: Causal Diagram depicting how variables are hypothetically related over time; Figure S2: Crude estimates of longitudinal mean profiles for Body Mass Index (BMI), Physical Activity (PA), Systolic Blood Pressure (SBP) and Diastolic Blood Pressure (DBP) across three visits (baseline, 6 and 12 months) in Mott Children’s Health Center patients in Flint, Michigan, 2021–2023.
