# Supplementary material for: Longitudinal Associations Between Fruit and Vegetable Prescription Program Participation and Pediatric Blood Pressure and Weight Status
Source: Nutrients. 2026 Oct 23;18(21):3476. doi: 10.3390/nu18213476 (PMC13765372; doi:10.3390/nu18213476)

Details on estimating the total exposure effects of the Fruit and Vegetable Prescription Program on study endpoints

Longitudinal analyses were conducted to estimate the total effects of the Fruit and Vegetable Prescription Program (FVPP) on study outcomes, focusing on prescription redemption. By total effects, we refer to both the indirect effect of the exposure at a given time point on a future outcome through future exposures (up to outcome timepoint) and its direct effect not passing through these future exposures. To be concrete, let y(t) denote the outcome at time t and x(t) the exposure at time just before t; that is x(t) refers to the exposure during the period [t-6, t) due to data being collected every 6 months. The total effect of x(t-a) on response y(t) includes both the indirect effect of x(t-a) on y(t) through future exposures x(t-a+6),… x(t) and its direct effect on y(t) not mediated through these future exposures. When a=0, there are no intermediate exposures and the total effect of x(t) on y(t) reduces to its direct effect. These would correspond to the direct effects of x(6) on y(6) and x(12) on y(12), representing the lag 1 effect. Lag 2 effect is the total effect of x(6), exposure between baseline and 6 months, on y(12) the outcome at 12 months. Evaluation of the total effects presents a challenge due to time varying confounding, which may include prior outcomes, FVPP exposures and time-varying covariates. Hence, to mitigate differences that might exist between the modalities of FVPP exposure at any timepoint, regression-based analyses that adjust for prior values of the exposure, outcome, time-varying and time invariant baseline covariates were considered. Selection of potential confounders was based on the endpoints. As an example for BMI, the following covariates were selected as potential confounders for the effect of the exposure to FVPP on BMI. Regardless of the lag, the basic sufficient set included the following variables: BMI method (chart vs RA), age of the child, sex of the child, race of the child, reason of clinic visit (sickness vs physical), child's health score, caregiver's education, caregiver's age, caregiver's BMI, participation in the Supplemental Nutrition Assistance Program (SNAP), participation in free/reduced school lunch programs, number of children under 18 in the household, number of months of exposure prior to entering in the study, and city of residence. These variables were supplemented with prior values of the exposure, outcome, time-varying and time invariant baseline covariates. All models were specified using parametric formulations (e.g., logistic and linear regression models depending on the nature of the outcomes) with selected time-varying confounders at each timepoint.

Supplemental Table S1: Missing data patterns among Mott Children's Health Center participants in Flint, Michigan, 2021-2023

Missing data patterns	Baseline	6 months	12 months	Freq	Percent
**1**	O	O	O	103	33.88
**2**	O	O	M	33	10.86
**3**	O	M	O	63	20.72
**4**	O	M	M	101	33.22
**5**	M	O	O	1	0.33
**6**	M	M	O	1	0.33
**7**	M	M	M	2	0.66

Note: O indicates subject was observed or present at the corresponding visit and M otherwise. Attrition patterns correspond to patterns 2 (O, O, M) and 4 (O, M, M).

Supplemental Table S2. Characteristics of study participants for clinics with an established fruit and vegetable prescription program (Clinics 1 and 2) compared to the newly initiated intervention clinic (Clinic 3) in Flint, Michigan, 2021-2023

**Characteristic**	Established intervention (Clinics 1 and 2)[a] (N=376)	Newly initiated intervention (Clinic 3)[a] (N=304)	p-value[b]
**Months of exposure - mean (range)**	40.70 (6.14)	7.76 (4.28)	<0.001
**Child age (yrs.) --- mean (SD)**	12.68 (2.51)	11.98 (2.39)	<0.001
**Caregiver age (yrs.) --- mean (SD)**	40.23 (8.80)	39.51(9.89)	0.32
**Child race --- no. (%)**			0.003
African American	294 (78.19)	204 (68.00)	
Other	82 (21.81)	96 (32.00)	
**Child biological sex --- no. (%)**			0.85
Female	194 (51.60)	159 (52.30)	
Male	182 (48.40)	145 (47.70)	
**Caregiver education --- no. (%)**			0.83
Less than high school (HS) degree	44 (11.76)	33 (10.93)	
HS or some college but no degree	243 (64.97)	193 (63.91)	
Trade, Associate degree or more	87 (23.26)	76 (25.17)	
**Caregiver BMI --- no. (%)**			0.02
Less than 30	106 (28.19)	113 (37.17)	
30 or more	186 (49.47)	143 (47.04)	
Unspecified	84 (22.34)	48 (15.79)	
**EBT/Bridge or SNAP --- no. (%)**			0.60
Yes	232 (61.70)	191 (63.67)	
No	144 (38.30)	109 (36.33)	
**Free/reduced school lunch --- no. (%)**			0.66
Yes	218 (57.98)	179 (59.67)	
No	158 (42.02)	121 (40.33)	
**City of residence --- no. (%)**			0.19
Flint	293 (77.93)	220 (73.58)	
Other	83 (22.07)	79 (26.42)	
**Caregiver's overall perceptions of child health care score---- mean (std)**	86.93 (14.12)	81.07 (18.05)	<0.001

BMI: body mass index, EBT: Electronic benefit transfer card, SNAP: Supplemental Nutrition Assistance Program

[a] Some cross-table cell counts do not add up to the marginal counts due to missing data in the demographic characteristics.
[b] p-values are generated using standard chi-square and student's t tests.

Supplemental Table S3: Means and standard deviations, and analytic sample sizes for food security, physical activity, fruit and vegetable intake, and prescription exposure across time in 3 pediatric clinics in Flint, Michigan, 2021-2023

Food security, physical activity, fruit and vegetable intake, prescription exposure	Established intervention (Clinics 1 and 2)		Newly initiated intervention (Clinic 3)					
	Baseline		Baseline		6 months		12 months	
	Mean (SD)	Sample size	Mean (SD)	Sample size	Mean (SD)	Sample size	Mean (SD)	Sample size
**Pediatric food security (PFS)**	1.47 (2.03)	221	1.35 (1.96)	142	1.68 (2.45)	74	1.68 (2.31)	103
**High PFS (PFS<=1)**	0.65 (0.48)	221	0.70 (0.46)	142	0.65 (0.48)	74	0.64 (0.48)	103
**Adult Food security (AFS)**	1.14 (1.71)	374	1.44 (1.94)	300	1.78 (2.18)	159	1.59 (2.08)	179
**High AFS (AFS<=1)**	0.69 (0.46)	374	0.64 (0.48)	300	0.59 (0.49)	159	0.63 (0.48)	179
**Physical activity**	22.48 (8.73)	366	19.80 (8.07)	296	22.43 (8.32)	136	21.75 (8.51)	171
**Fruit**	1.47 (1.32)	374	1.35 (1.11)	300	1.57 (1.13)	137	1.51 (1.11)	168
**Whole Fruits**	0.70 (0.72)	374	0.65 (0.65)	300	0.79 (0.65)	137	0.69 (0.66)	168
**Vegetable (excludes legumes)**	1.04 (0.94)	374	1.09 (0.93)	300	1.19 (0.94)	137	1.05 (0.81)	168
**Vegetable (excludes potatoes and legumes)**	0.67 (0.00)	374	0.72 (0.64)	300	0.79 (0.68)	137	0.68 (0.57)	168
**Legumes**	0.05 (0.12)	374	0.06 (0.11)	300	0.07 (0.13)	137	0.04 (0.09)	168
**Other fruit**	0.13 (0.22)	374	0.12 (0.18)	300	0.15 (0.21)	137	0.13 (0.17)	168
**Fruit juice**	0.64 (0.72)	374	0.58 (0.63)	300	0.63 (0.67)	137	0.70 (0.66)	168
**Whole grains**	0.54, (0.65)	374	0.56 (0.64)	300	0.59 (0.55)	137	0.54 (0.51)	168
**Prescriptions received[a]**	-	-	0.58 (0.98)	302	1.90 (1.11)	302	0.83 (1.11)	302
**#Prescriptions received > 0**	-	-	0.36 (0.48)	302	1.00 (0.00)	302	0.48 (0.50)	302
**Prescriptions redeemed[a]**	-	-	0.19 (0.61)	302	0.55 (0.94)	302	0.26 (0.64)	302
**#Prescriptions redeemed > 0**	-	-	0.13 (0.33)	302	0.35 (0.48)	302	0.19 (0.39)	302

[a]Prescriptions received or redeemed correspond to the numbers of prescriptions accumulated in the last 6 months, prior to the visit. For example, the numbers at 6 month visit correspond to the numbers of prescriptions between baseline and 6 months, with 6 months excluded.

Supplemental Table S4: Means and standard deviations, and analytic sample sizes for clinical outcomes among patients from 3 pediatric clinics in Flint, Michigan, 2021-2023

Clinical outcomes	Established intervention (Clinics 1 and 2)		Newly initiated intervention (Clinic 3)					
	Baseline		Baseline		6 months		12 months	
	Mean (SD)	Sample size	Mean (SD)	Sample size	Mean (SD)	Sample size	Mean (SD)	Sample size
**BMI Percentile (BMI %)**	76.04 (27.34)	374	75.90 (28.47)	296	73.45 (29.88)	141	74.94 (27.58)	192
**BMI %> 95%**	0.40 (0.49)	374	0.41 (0.49)	296	0.38 (0.49)	141	0.39 (0.49)	192
**Systolic BP[b] % (SBP%)**	59.69 (24.33)	373	73.72 (23.14)	280	69.99 (21.99)	136	67.57 (22.89)	176
**SBP% > 90%**	0.09 (0.28)	373	0.32 (0.47)	280	0.18 (0.39)	136	0.22 (0.42)	176
**Diastolic BP % (DBP%)**	70.49 (21.11)	373	76.04 (20.56)	280	75.52 (18.63)	136	71.39 (19.51)	176
**DBP% > 90%**	0.19 (0.39)	373	0.30 (0.46)	280	0.28 (0.45)	136	0.19 (0.39)	176
**Overall elevated BP**	0.25 (0.43)	376	0.44 (0.50)	300	0.38 (0.49)	127	0.33 (0.47)	165

BMI: body mass index, BP: blood pressure

Supplemental Table S5: Analysis evaluating the lag 1 and 2 effects of prescription redemption on physical activity, food security and fruit and vegetable intake in Mott Children's Health Center patients in Flint, Michigan, 2021-2023

Food security, fruit and vegetable intake and physical activity	Effects[a]			
	Lag 1 (short term)		Lag 2 (long term)	
	Estimate (CI)	p-value[c]	Estimate (CI)	p-value[c]
**Food security[b]**				
•Youth high food security	0.82 (0.41, 1. 64)	0.57	0.94 (0.45, 1.98)	0.88
•Household high food security	0.75 (0.45, 1.24)	0.26	0.60 (0.37, 0.99)	0.05
**Youth fruit and vegetable intake**				
•Total fruits	-0.06 (-0.30, 0.18)	0.62	-0.05 (-0.30, 0.20)	0.70
•Total whole fruits	-0.07 (-0.21, 0.08)	0.37	-0.01 (-0.15, 0.14)	0.91
•Total vegetables	-0.07 (-0.23, 0.09)	0.41	-0.13 (-0.29, 0.04)	0.13
•Vegetable excl. potatoes/legumes	-0.04 (-0.16, 0.08)	0.48	-0.08 (-0.21, 0.04)	0.19
•Total legumes	-0.01 (-0.03, 0.01)	0.56	-0.02 (-0.04, -0.003)	0.02
•Other fruits	-0.003 (-0.04, 0.03)	0.89	-0.001 (-0.04, 0.04)	0.98
•Total fruit juice	0.005 (-0.13, 0.14)	0.95	0.006 (-0.16, 0.17)	0.95
**Physical activity**				
•Raw score	0.60 (-1.04, 2.24)	0.47	0.27 (-1.72, 2.26)	0.79

[a] Estimates of total effects of FVPP for lag 1 and 2 are computed taking into account the presence of time-dependent confounding, by controlling for prior exposures, prior outcomes and prior time-varying covariates. Confidence intervals are generated using sandwich-based standard errors.
[b] Effect estimates are on the odds ratio scale.
[c] P-values are generated using z tests from GEE models.

Additional information about sensitivity analyses

Handling of missing RA measurements of height and weight

During the three study visits, research assistant (RA) measurements of height and weight were unavailable for 25 (3.73%), 105 (74.47%) and 52 (27.08%), respectively at baseline, 6-month and 12-month follow-up visits. Results of the available case analysis that uses clinic measurements recorded in medical records in place of missing RA measurements were presented in the manuscript. A sensitivity analysis was also conducted by not replacing unavailable RA measurements with clinic measurements recorded in medical records. Both the classical longitudinal analysis results and the lag effect inference results are similar (qualitatively) to the analysis that incorporates the RA measures.

Supplemental Table S6: Sensitivity analysis for contrasts evaluating the longitudinal trend for Body Mass Index (BMI) percentile (mean difference) and obesity (odds ratios); adjusted estimates and confidence intervals in Mott Children's Health Center patients in Flint, Michigan, 2021-2023

Clinical outcomes	Longitudinal comparisons[a]			
	6 months versus baseline	12 months versus baseline	12 months versus 6 months	Overall Trend p-value[c]
**Child weight status**				
•BMI percentiles (%)	0.53 (-3.87, 4.92)	-3.31 (-5.23, -1.39)	-3.84 (-7.96, 0.28)	0.001
•Obesity (BMI % >95%)[b]	0.58 (0.30, 1.13)	0.86 (0.65,1.14)	1.49 (0.77, 2.88)	0.21

BMI: body mass index

[a]The contrast estimates of trend are generated by controlling the following confounders: child's age, child's sex, child's race (African American versus others), caregiver's education, caregiver's age (age < 38 years vs. age ≥ 38 years), caregiver's BMI (BMI < 30, BMI ≥30, unspecified), participation of any member of the household to any supplemental nutrition assistance program or Free/reduced school lunch (yes vs. no), city of residence (Flint vs. other) and caregiver's overall perceptions of child health care (score <90, vs. score ≥ 90).
[b]Estimates on the odds ratio scale.
[c] P-values are derived from F test statistics.

Supplemental Table S7: Sensitivity analysis on effects evaluating the lag 1 and 2 effects of prescription redemption on Body Mass Index (BMI) percentiles and obesity (estimates, confidence intervals in parenthesis and p-values) in Mott Children's Health Center patients in Flint, Michigan, 2021-2023

Clinical outcomes	Effects[a]			
	Lag 1 (short term)		Lag 2 (long term)	
	Estimate (CI)	p-value[c]	Estimate (CI)	p-value[c]
**Child weight status**				
•BMI percentiles (%)	-0.68 (-8.24, 6.88)	0.86	-12.59 (-20.05, -5.14)	<0.001
•Obesity (BMI % >95%)[b]	0.65 (0.31, 1.39)	0.27	0.15 (0.60, 0.38)	<0.001

BMI: body mass index

[a] Estimates of total effects of FVPP for lag 1 and 2 are computed taking into account the presence of time-dependent confounding, controlling for prior exposures, prior outcomes and prior time-varying covariates. Confidence intervals are generated using sandwich-based standard errors.
[b] Effect estimates are on the odds ratio scale.
[c] P-values are generated using z tests from GEE models

Supplemental Figure S1: Causal Diagram depicting how variables are hypothetically related over time

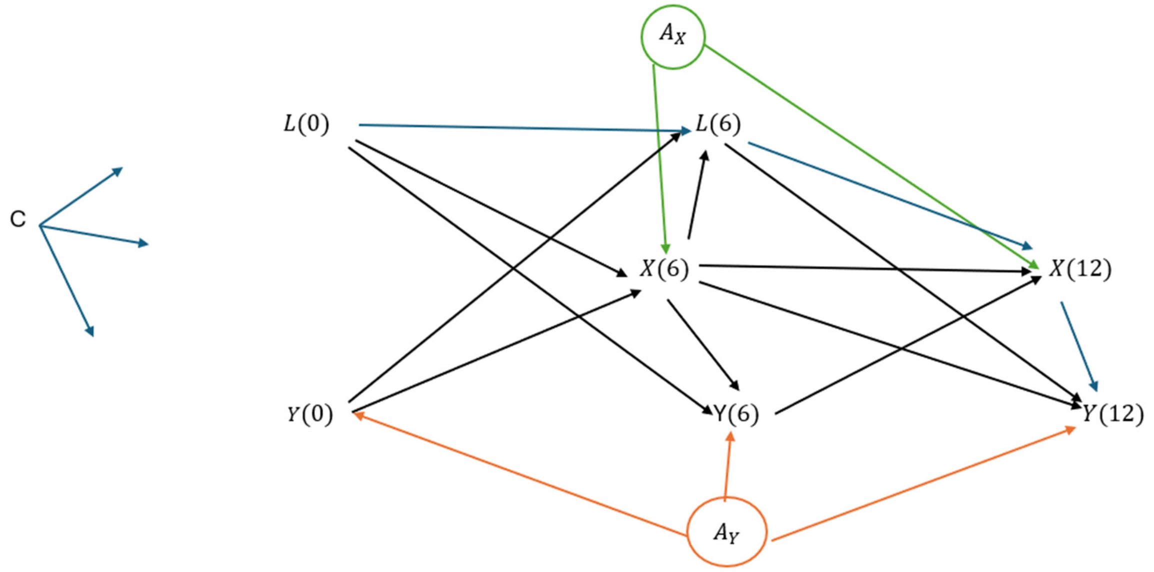

Figure S1 depicts how variables are hypothetically related over time in our causal model with data at timepoints t=0 (Baseline), t=6 (6 months visit) and t=12 (12 months visit). In this causal diagram, Y(t) t=0, 6, 12, denotes the outcome (e.g., BMI) at time t and X(t), t=6, 12, the binary exposure (redeeming y/n at least 1 prescription) at time just before t; that is X(t) refers to the exposure during the period [t-6, t) due to data being collected every 6 months. L(t) represents the set of time varying confounders at time t, t=0, 6. C represents the set of baseline time invariant confounders such as child sex/gender, race, residence, Caregiver's baseline BMI. A_X and A_Y are unobserved random effects that tie together the serial exposures or serial outcomes over time, such that upon conditioning on each of these random effects the associated exposures and outcomes will be independent. From this model, L(t) are confounders for the association between X(t) and Y(t) but on the pathway from X(t-6) and Y(t). Typical variables in L(t) are caregiver's overall perceptions of pediatric health care, and participation of any member of the household to any supplemental nutrition assistance program or Free/reduced school lunch (yes vs. no).

The causal inference analysis requires three conditions that must be met for causal effect estimates to have valid interpretations: consistency, positivity, and exchangeability. Consistency refers to the design and delivery of an intervention that is well-defined and implemented in the same way across study subjects. Positivity is the potential for all study population individuals to receive the intervention. Exchangeability is the state in which the two comparisons groups are interchangeable, or in other words there is no unmeasured confounding. Due to the time-varying nature of our exposure (redeeming at least a prescription, Y/N), these conditions need to be met

for the two exposure periods (baseline to 6 months and 6 months to 12 months) for a reliable estimation of lag1 and lag 2 causal effects.

A working example: Evaluation of the lag 2 effect of FVPP redemption on the BMI; that is the total (direct and indirect) effect of X(6) on Y(12). Binary X(6) indicates whether the participant has redeemed at least 1 prescription between baseline and 6 months, and Y(12) is the BMI percentile at 12 months. The indirect effects X(6) on Y(12) is via the pathways X(6) → X(12)→ Y(12) and X(6) → L(6) → X(12)→ Y(12), and the direct effect is via the path X(6) → Y(12) and X(6) → L(6)→ Y(12). C and L(0) are the confounders for the association X(6) and Y(12). C represents the set of baseline time invariant confounders such as child sex/gender, race, residence, Caregiver's baseline BMI. L(0) is the set of caregiver's overall perceptions of pediatric health care, and participation of any member of the household to any supplemental nutrition assistance program or Free/reduced school lunch (yes vs. no), at time=0 (baseline).

Supplemental Figure S2: Crude estimates of longitudinal mean profiles for Body Mass Index (BMI), Physical Activity (PA), Systolic Blood Pressure (SBP) and Diastolic Blood Pressure (DBP) across three visits (baseline, 6 and 12 months) in Mott Children's Health Center patients in Flint, Michigan, 2021-2023

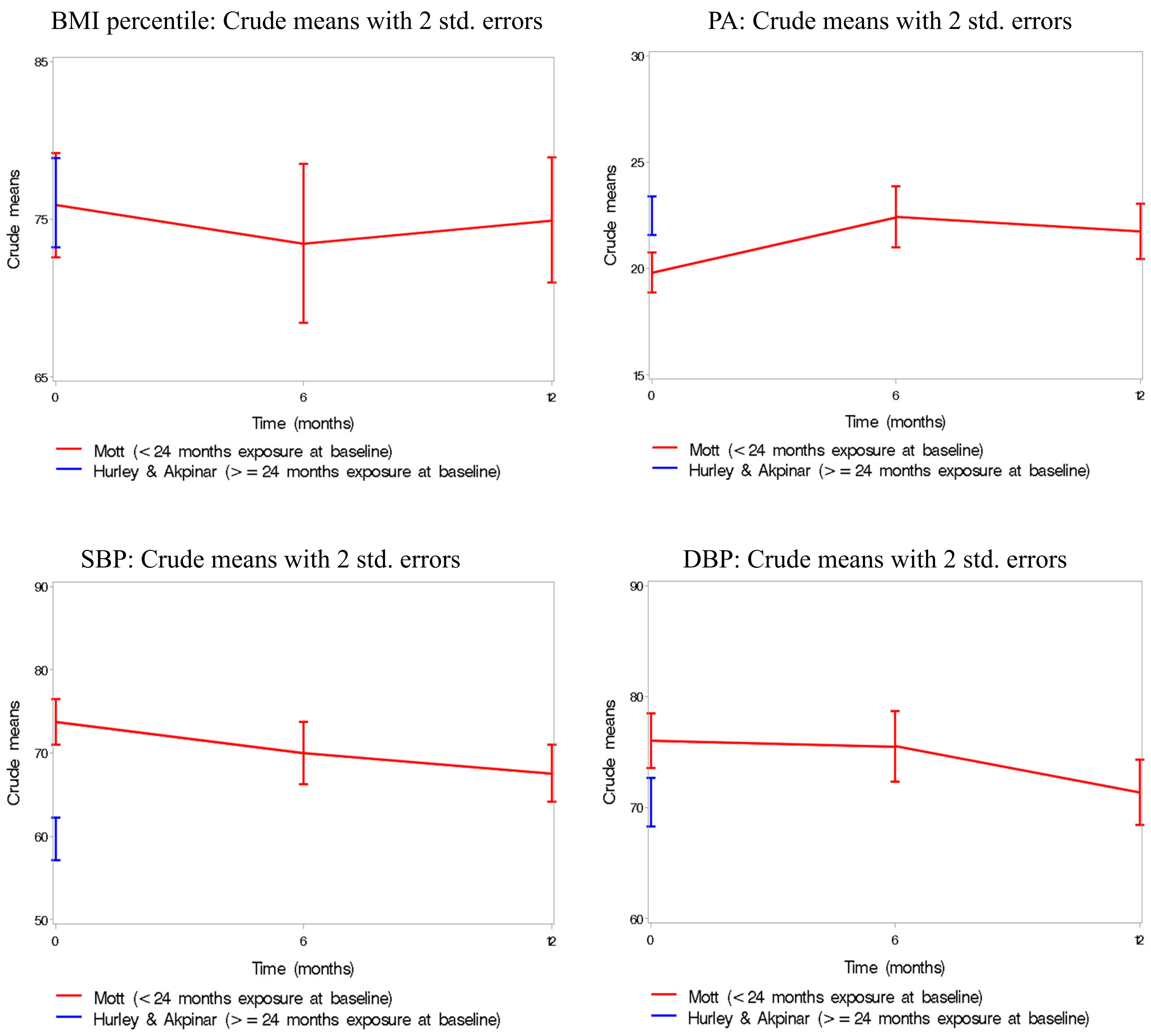

Supplement: Supplementary file 1 [file nutrients-18-03476-s001.zip › nutrients-4598757-supplementary.pdf]
